# Comparison of Ordinary Cannulated Compression Screw and Double-Head Cannulated Compression Screw Fixation in Vertical Femoral Neck Fractures

**DOI:** 10.1155/2020/2814548

**Published:** 2020-12-30

**Authors:** Yuelei Zhang, Chao Yan, Lecheng Zhang, Wei Zhang, Gang Wang

**Affiliations:** ^1^Department of Orthopedics, The First Affiliated Hospital of Anhui Medical University, Hefei 230000, China; ^2^Department of Orthopedic Surgery, Shanghai Jiao Tong University Affiliated Sixth People's Hospital, 600 Yishan Road, Shanghai 200233, China

## Abstract

**Background:**

The treatment of vertical femoral neck fractures in young patients remains a challenge. This study is aimed at comparing ordinary cannulated compression screw (OCCS) and double-head cannulated compression screw (DhCCS) fixation in vertical femoral neck fractures both clinically and biomechanically.

**Materials and Methods:**

Clinically, the radiographs of 81 patients with Pauwel's III femoral neck fractures, including 54 fractures fixed with three parallel OCCSs and 27 fractures fixed with three parallel DhCCSs, were reviewed retrospectively. Complications consisting of fixation failure (screw loosening, obvious fracture displacement, varus deformity, or femoral neck shortening), bony nonunion, and avascular necrosis (AVN) were determined. Biomechanically, twenty synthetic femur models of vertical femoral fractures with an 80° Pauwel's angle were divided into two groups and subsequently fixed with three parallel OCCSs or DhCCSs. All specimens were tested for axial stiffness, load to 5 mm displacement, and a maximum load to failure with a loading rate of 2 mm/min.

**Results:**

Clinically, 22 fractures in the OCCS group experienced fixation failure, including 19 screw loosening, 18 femoral neck shortening, 14 varus deformities, and 8 obvious fracture displacements, whereas only 4 fractures experienced fixation failure in the DhCCS group, including 3 screw loosening, 3 femoral neck shortening, 3 varus deformities, and 1 obvious fracture displacement. Additionally, 11 fractures in the OCCS group exhibited nonunion, whereas only 3 in the DhCCS group exhibited nonunion. Nine fractures with AVN were noted in the OCCS group, whereas only 1 was observed in the DhCCS group. Biomechanically, the axial stiffness of the DhCCS group was greater than that of the OCCS group (154.9 ± 6.81 vs. 128.1 ± 7.41 N/mm), and the load to 5 mm displacement was also significantly greater in the DhCCS group (646.1 ± 25.87 vs. 475.8 ± 21.46 N). Moreover, the maximum load to failure in the DhCCS group exhibited significant advantages compared with that of the OCCS group (1148 ± 39.47 vs. 795.9 ± 51.39 N).

**Conclusion:**

Our results suggested that using three DhCCSs improved the outcome of vertical femoral neck fractures compared to three OCCSs, offering a new choice for the treatment of femoral neck fracture.

## 1. Introduction

Femoral neck fracture in young adults is usually the result of high energy, and salvage of the femoral head with anatomic reduction and stable fixation is preferred [1, 2]. Cannulated screws have become the most common fixation device given their linear dynamic compression during weight bearing, less invasive surgery, less blood loss, and shorter hospital stay [3]. However, for more vertical femoral neck fractures that are axial and rotationally unstable, strong shear forces across the hip frequently lead to fixation failure when fixed with ordinary cannulated compression screw (OCCS) with overall complication rates ranging from 20% to 86% [4, 5].

No standard internal fixation has been clinically proven to be superior for vertical fractures, although the addition of a cross screw or combination of a sliding hip screw and an additional antirotation screw has been recommended [6, 7]. However, higher nonunion and AVN rates were reported by Parker et al. [8]. In addition, a medial buttress plate was proposed to augment the fixation of ordinary cannulated screws, which still requires longer follow-up and a larger sample size [3].

The double-head cannulated compression screw (DhCCS) has been used for fractures of many sites with favorable clinical results reported in the literature [9, 10], whereas rare outcomes of DhCCS, which functions through linear compression and local locking with the proximal lateral femoral cortex, in femoral neck fracture were reported. The purpose of this study is to compare the clinical and mechanical outcomes of two types of cannulated screws, OCCS and DhCCS, when used in vertical shear femoral neck fractures.

## 2. Materials and Methods

### 2.1. Patients and Methods

This research was approved by the IRB of the authors' affiliated institutions. The office approved that verbal consent obtained by telephone was required because this study is retrospective, and all the radiographs and data required in this study were recorded in the case system of the hospital. From January 2017 to December 2018, 417 femoral neck fractures in Shanghai Jiao Tong University Affiliated Sixth People's Hospital treated with internal fixation were retrospectively studied. The exclusion criteria were as follows: (1) fractures treated with other internal fixation constructs rather than three parallel cannulated screws, including four cannulated screws with a cross screw, a sliding hip screw, and an additional antirotation screw; (2) femoral head fractures together with other ipsilateral or contralateral lower limb fractures; (3) fractures with opening reduction; (4) patients without adequate reduction postoperatively; (5) pathological fractures; (6) femoral neck fractures with Pauwel's angle less than 50 degrees in standard anteroposterior pelvic radiographs before or after operation; and (7) fractures with a follow-up period shorter than 9 months. Adequate reduction was considered when a femoral neck angle was <10° varus or <15° valgus and the displacement between the fracture fragments was less than <3 mm on AP and lateral radiographs compared to the contralateral hip on an anteroposterior (AP) pelvis radiograph [11]. Patients and/or their families were informed and approved that data from the cases would be submitted for publication.

**Figure 1:**
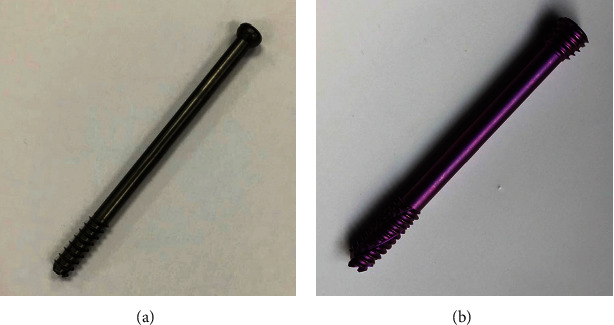
Application of OCCS and DhCCS in femoral neck fractures. (a) The ordinary cannulated compression screw. (b) Double-head cannulated compression screw.

**Figure 2:**
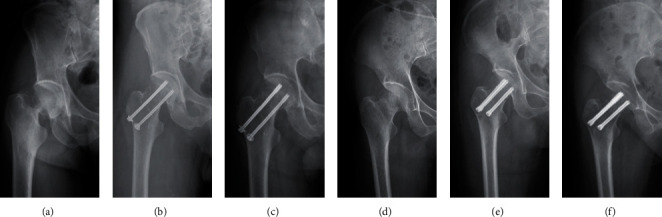
Typical radiographs of femoral neck fractures with success in the OCCS group and the DhCCS group. (a–c) Radiographs of injury, postoperation, and 6 months after operation in the OCCS group. (d–f) Radiographs of injury, postoperation, and 6 months after operation in the DhCCS group.

Finally, 81 patients with vertical femoral neck fractures (Pauwel′s angle > 50°) whose operation was performed by three surgeons were included in this study. There were 10 Garden III fractures and 71 Garden IV fractures. All fractures were treated with three parallel cannulated compression screws, including 54 fractures (6 Garden III and 48 Garden IV) fixed with OCCS (7.3 mm, DePuy Synthes Co., New Jersey, USA) and 27 fractures (4 Garden III and 23 Garden IV) fixed with DhCCS (7.5 mm, Integra LifeSciences Co., Lyon, France) (Figures 1 and 2). All patients followed a similar postoperative protocol as follows: no weight bearing of the affected limb within 3 months, partial weight bearing after bone union, and total weight bearing until 6 months postoperatively.

### 2.2. Radiographic Analysis

Anteroposterior and lateral radiographs were obtained at 6 weeks, 3 months, 6 months, 9 months, and 12 months after the surgery and at any time when pain appeared at the injured hip. Fixation failure was defined as screw loosening, obvious fracture displacement (>5 mm), varus deformity (>10°), or femoral neck shortening (FNS) (>10 mm vertically) compared with preliminary fixation [12–14]. Bone nonunion was described as lack of any healing on plain or CT radiographs within 9 months. Avascular necrosis was identified as the appearance of subchondral sclerosis or the presence of segmental collapse [14]. All radiographs were gathered and analyzed by the authors with a consensus.

### 2.3. Specimen Preparation

Twenty same shaped left side synthetic femur models (FZ001, ENOVO, Shanghai, China) were equally divided into two groups, and all specimens were predrilled under C-arm fluoroscopic guidance prior to osteotomy to facilitate anatomic reduction and ideal implant positioning. The vertical fracture model was osteotomized with an oscillating saw at an 80° angle to the horizontal line and then repaired with three parallel OCCSs (the OCCS group with 10 models) or DhCCSs (the DhCCS group with 10 models) with an inverted triangle configuration. All the models were osteotomized horizontally at the inferior segment with the same height from the apex of the greater trochanter.

### 2.4. Biomechanical Analysis

**Figure 3:**
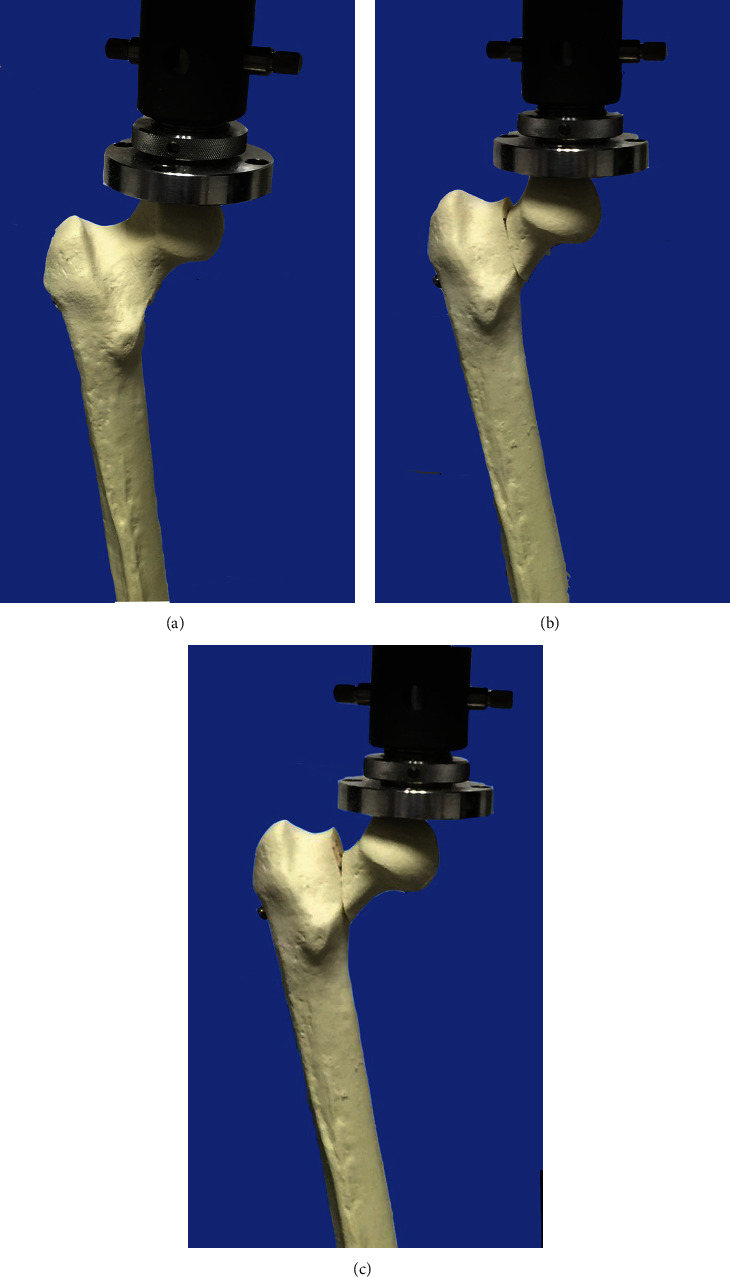
Biomechanical process of a femur synthetic bone model fixed with cannulated screws.

Biomechanical analysis was performed using an Instron test system (Instron, Norwood, MA, USA). Two magnets were placed on the femoral head and proximal femur to record the displacement of the femoral head with axial loading. Distally, the femur was fixed to the base of a mechanical tester with dental powder at a shaft adduction angle of 7° to simulate normal walking. All tests were conducted with an axial compressive loading, which was performed with a loading rate of 2 mm/min. The maximum load for failure was defined by the destructive construct failure followed by a marked decrease in the applied load value [15, 16] (Figure 3).

### 2.5. Statistical Analysis

The data, including the gender and hip affected, were compared using the chi-square test. All measurement data, including the age, Pauwel's angle, follow-up period, axial load, and stiffness between the two groups, were expressed as the mean ± standard deviation and compared using the independent sample *t*-test. Statistical analysis was performed with SPSS, and significant differences were considered when *p* < 0.05.

## 3. Results

Initially, we performed a retrospective study. The mean age of patients in the OCCS group was 49.1 ± 12.5 years with no significant difference from the patients in the DhCCS group (45.8 ± 14.2). In total, 30 (55.6%) males were included in the OCCS cohort, and 17 males (63%) were included in the DhCCS cohort. In total, the right hip was affected in 31 patients (57.4%) in the OCCS group and 16 (59.3%) in the DhCCS group. The mean follow-up period of patients treated with OCCS was 18.15 ± 6.45 months, which is similar to that in the DhCCS group (18.48 ± 3.47) (Table 1).

**Figure 4:**
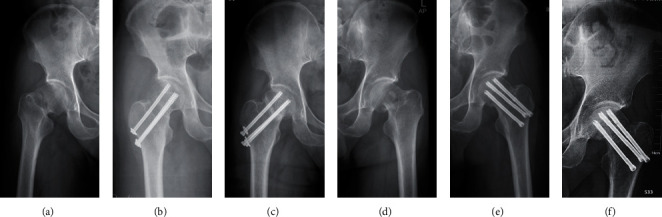
Typical radiographs with fixation failures of vertical femoral neck fractures. (a–c) Radiographs of injury, postoperation, and 6 months after operation in the OCCS group with screw loosening and FNS. (d–f) Radiographs of injury, postoperation, and 6 months after operation in the DhCCS group with screw loosening, varus deformity and FNS.

**Figure 5:**
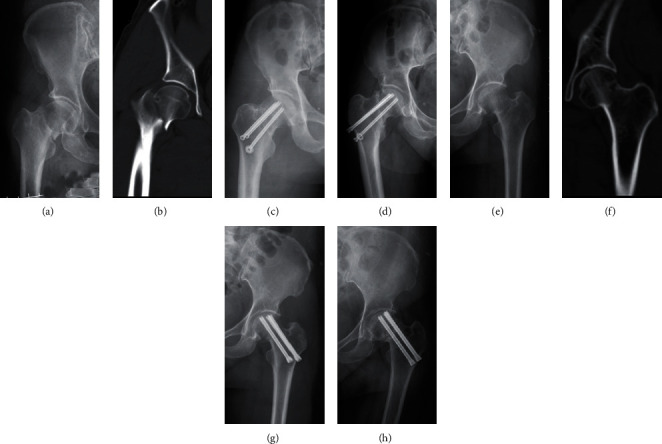
Typical radiographs with nonunion of vertical femoral neck fractures. (a–d) Radiographs of injury, postoperation, and 9 months after operation in the OCCS group with screw with nonunion. (e–h) Radiographs of injury, postoperation, and 9 months after operation in the DhCCS group with nonunion.

For these fractures, no obvious difference was found between the Pauwel's angle in the two groups (58.64 ± 5.23 in the OCCS group vs. 57.54 ± 5.62 in the DhCCS group), which were measured on the standard anteroposterior pelvic X-ray before or after the surgery (Table 1). However, distinctly different radiographic outcomes were observed between the groups. Twenty-two (40.74%) fractures in the OCCS group experienced fixation failure, including 19 (35.19%) screw loosening, 18 (33.33%) FNS (>10 mm vertically), 14 (25.93%) varus deformity (>10°), and 8 (14.81%) obvious fracture displacement (>5 mm), given that different types of fixation failure always appear together, whereas 4 (14.81%) fractures exhibited fixation failure in the DhCCS group, including 3 (11.11%) screw loosening, 3 (11.11%) FNS, 3 (11.11%) varus deformity (>10°), and 1 (3.70%) obvious fracture displacement (>5 mm) (Figure 4). Additionally, 11 (20.37%) fractures in the OCCS group had nonunion, whereas only 3 (11.11%) fractures in the DhCCS group exhibited nonunion (Figure 5). Furthermore, we observed 9 (16.67%) AVNs in the OCCS group and only 1 (3.7%) AVN in the DhCCS group (Table 2). Furthermore, DhCCS provided lower fixation failure rate than OCCS in Garden IV fractures, although no difference was observed in Garden III fractures (Tables 3 and 4).

**Figure 6:**
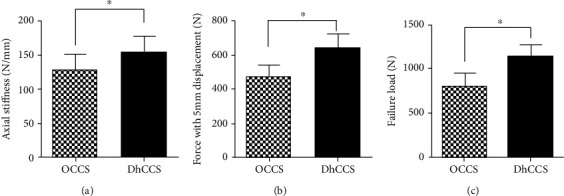
Biomechanical results in the OCCS group and the DhCCS group. Data are shown as the mean ± standard deviation. ^∗^Significant difference between the two groups (*p* = 0.015 for axial stiffness, *p* < 0.0001 for force with 5 mm displacement, and *p* < 0.0001 for failure load).

The biomechanical results showed that the DhCCS group exhibited better biomechanical stability than the OCCS group, especially with respect to the maximum load to failure (1148 ± 39.47 vs. 795.9 ± 51.39 N) and the load to 5 mm displacement (646.1 ± 25.87 vs. 475.8 ± 21.46 N). Moreover, the axial stiffness of the DhCCS group was greater than that of the OCCS group (154.9 ± 6.81 vs. 128.1 ± 7.41 N/mm) (Figure 6).

## 4. Discussion

A high rate of complications with vertical femoral neck fractures has been reported by many authors, especially for fractures fixed with OCCS. In this study, we compared the fixation outcomes of OCCS and DhCCS both clinically and biomechanically. Our results showed that DhCCS had lower fixation failure rates and stronger stability than OCCS, indicating that DhCCS is a promising fixation construct for vertical femoral neck fractures.

Cannulated compression screws have been introduced in the treatment of femoral neck fractures for many years, and the advantages of these screws include less tissue invasiveness, less blood loss, shorter hospital stay, and reduced operation time. However, the method of dynamic compression also increased the rate of fixation failure, especially for vertical shear femoral neck fractures, and high rates of complications, including displacement, screw loosening, femoral neck shortening, varus deformity, and nonunion, have been reported by many authors [11, 17, 18]. Our retrospective study also strongly confirmed this viewpoint. Therefore, some scholars have proposed the use of different types of cannulated compression screws to improve their fixation effect. Filipov et al. [19] introduced a biplane double-supported screw fixation method with three OCCSs, and their study showed that this method enhanced femoral neck fracture fixation strength and revealed excellent clinical outcomes. Zhang et al. [20] introduced a new configuration of cannulated screw fixation with two headless cannulated compression screws (HCCS) plus an OCCS in parallel, which demonstrated significant advantages compared with OCCS alone both biomechanically and clinically. Liu et al. [21] further demonstrated that the modified fixation of cannulated screws could improve the biomechanical performance and buttress the femoral head fragment better than OCCS in femoral neck fractures with comminuted posteromedial cortex. Their biomechanical study showed that [22] HCCS performed with better biomechanical stability than OCCS in the treatment of vertical femoral neck fracture, especially with a Pauwel's angle of 70°. DhCCS in this study also provided an encouraging outcome with a smaller failure rate in patients and a better stability in models compared with OCCS.

Sliding implants, such as OCCS, can lead to FNS, which causes abductor muscle weakness as a result of a decreased abductor moment arm [23]. Chen et al. [24] reported that FNS occurred in 41.8% of elderly patients after fracture fixation with multiple cancellous sites and negatively affected postoperative joint function without affecting fracture union, whereas parallel implantation exhibited an increased shortening incidence compared with strong oblique implantation. To minimize this phenomenon, Weil et al. [23] introduced the use of fully threaded screws and demonstrated that these screws decreased FNS after fixation of femoral neck fractures compared with OCCS. Similarly, the application of HCCS also decreased FNS, as previously reported [20]. DhCCS exerts threads at the proximal and distal ends, and the diameter of the distal thread portion is greater than that at the proximal end with both threads during insertion engaging with the bone. Moreover, the thread pitches at the proximal end are greater than those at the distal end. This thread profile can produce larger compression between fracture fragments than OCCS when the screw is driven. Furthermore, the interlocking of the distal thread portion and the lateral femur cortex maintains the lengths of the femoral neck after reduction, limiting shortening of the femoral neck. Similarly, this interlocking effect provides sufficient support on the lateral femoral cortex to significantly reduce the rate of varus deformity compared to OCCS [25], and the occurrence of screw loosening was also reduced in DhCCS as observed in our retrospective study.

Fracture union depends on the stability between fractures. For vertical femoral neck fractures fixed with OCCS, the strong shear force leads to displacement and finally nonunion due to screw sliding. For DhCCS, the special thread design allowed it to obtain more stable support to counter against the shear force in vertical femoral neck fracture, resulting in reduced fracture displacement and nonunion. In addition, the increase in screw diameter will also increase the strength of fracture fixation as demonstrated in our mechanical analysis.

AVN is one of the most common complications of femoral neck fractures due to damage to the special blood supply of the femoral head. The overall occurrence of AVN is as high as 20-30% after fractures [26]. Although a lower rate of AVN of DhCCS than OCCS was observed in our study, symptomatic AVN may appear as late as 6 years post injury, and 29% of patients with AVN exhibited no significant symptoms [27]. Therefore, due to the limited and nonscheduled follow-up of this study, the effect of DhCCS on AVN still requires further study.

Several limitations of this study, including the retrospective nature, the small number of patients available for radiographic follow-up, synthetic bones rather than fresh-frozen human cadaveric specimens, no cyclic tests performed to elucidate the fatigue-like behavior of the constructs and no establishment of femoral neck shortening model were acknowledged. Nevertheless, we clearly demonstrated the advantages of DhCCS for vertical femoral neck fractures; of course, a larger-scale study is still needed to strengthen these conclusions.

## 5. Conclusion

In summary, DhCCS may exert more advantages than OCCS in the treatment of vertical femoral neck fractures, including stronger fixation stability, lower rate of fixation failure, and nonunion. However, further investigations with an increased number of patients are needed in the future.

## Figures and Tables

**Table 1:** Patient demographics and clinical characteristics.

	OCCS	DhCCS	*p*
Number	54	27	—
Age (years old)	49.1 ± 12.5	45.8 ± 14.2	0.372^a^
Sex (M/F)	30/24	17/10	0.524^b^
Hip (R/L)	31/23	16/11	0.874^b^
Follow-up period (month)	18.15 ± 6.45	18.48 ± 3.47	0.803^a^
Pauwel's angle (°)	58.64 ± 5.23	57.54 ± 5.62	0.807^a^

Data are expressed as the mean ± standard deviation unless stated otherwise. ^a^Independent sample *t*-test. ^b^Chi-square test.

**Table 2:** Radiographic analysis of fractures fixed with OCCS and DhCCS.

	OCCS (%)	DhCCS (%)	*p*
Number	54	27	—
Fixation failure	22 (40.74)	4 (14.81)	0.018
Screw loosening	19 (35.19)	3 (11.11)	0.022
Femoral neck shortening	18 (33.33)	3 (11.11)	0.031
Varus deformity	14 (25.93)	3 (11.11)	0.123
Fracture displacement	8 (14.81)	1 (3.7)	0.259
Nonunion	11 (20.37)	3 (11.11)	0.365
ONFH	9 (16.67)	1 (3.70)	0.153

**Table 3:** Radiographic analysis of Garden III fractures fixed with OCCS and DhCCS.

	OCCS (%)	DhCCS (%)	*p*
Number	6	4	—
Fixation failure	2 (33.33)	0 (0)	0.467
Nonunion	1 (16.67)	1 (33.33)	1.000
ONFH	1 (16.67)	0 (0)	1.000

**Table 4:** Radiographic analysis of Garden IV fractures fixed with OCCS and DhCCS.

	OCCS (%)	DhCCS (%)	*p*
Number	48	23	—
Fixation failure	20 (41.67)	4 (17.39)	0.043
Nonunion	10 (20.83)	2 (8.70)	0.313
ONFH	8 (16.67)	1 (4.35)	0.254

## Data Availability

The data used to support the findings of this study are available from the corresponding author upon request.
